# Supplementary material for: Effects of reduced dissolved oxygen concentrations on physiology and fluorescence of hermatypic corals and benthic algae
Source: PeerJ. 2014 Jan 2;2:e235. doi: 10.7717/peerj.235 (PMC3898309; doi:10.7717/peerj.235)
Supplement: Table S1 — Results from two-way ANOVA analysis with the main effects of oxygen treatment (3-levels) and species (2-levels) and their interaction on data measured using both coral and algae (fluorescence analyses were excluded of this analysis because not shared by both species). Statistically significant effects are shown in bold. [file peerj-02-235-s001.docx]

| **Parameter** | **Source** | **degree of freedom** | **sum of squares** | **F Ratio** | **Prob > F** |
| --- | --- | --- | --- | --- | --- |
| Oxygen production | oxygen treatment | 2 | 0.311550 | 2.7263 | 0.0817 |
|  | species | 1 | 0.039336 | 0.6884 | 0.4132 |
|  | species*oxygen treatment | 2 | 0.402272 | 3.5202 | **0.0423** |
| Maximum QY | oxygen treatment | 2 | 66,107.722 | 5.3293 | **0.0105** |
|  | species | 1 | 31,093.444 | 5.0133 | **0.0327** |
|  | species*oxygen treatment | 2 | 55,893.056 | 4.5059 | **0.0194** |
| Effective QY | oxygen treatment | 2 | 8,177.556 | 1.8000 | 0.1827 |
|  | species | 1 | 19,413.778 | 8.5467 | **0.0065** |
|  | species*oxygen treatment | 2 | 11,308.222 | 2.4892 | 0.1000 |
