# Supplementary material for: Effects of reduced dissolved oxygen concentrations on physiology and fluorescence of hermatypic corals and benthic algae
Source: PeerJ. 2014 Jan 2;2:e235. doi: 10.7717/peerj.235 (PMC3898309; doi:10.7717/peerj.235)
Supplement: Table S2 — Contribution (%) that each of the studied factors (oxygen treatment, experimental time, individual repeat, and individual repeat variation over time) has on the metrics of organism health measured from the coral and algae. The residual indicates fraction of the contribution that remains unexplained by the present factors. Each factor is associated with a different degree of freedom (DF); statistically significant effects are shown in bold (p < 0.05) and marked with an asterisk when p < 0.01 (from Repeated Measures ANOVA). [file peerj-02-235-s002.docx]

| Parameter | **Factor** | **DF** | **% effect on algae** | **% effect on corals** |
| --- | --- | --- | --- | --- |
| O_2_ production | Treatment | 2 | **12.5** | 14.1 |
|  | Time | 15 | **24.2** | **37.6** |
|  | Repetition | 4 | **15.5*** | 1.5 |
|  | Repetition * Time | 8 | 3.0 | 9.3 |
|  | Residual | 60 | 44.9 | 37.5 |
| Maximum QY | Treatment | 2 | 9.2 | **25.0*** |
|  | Time | 15 | **47.5** | **18.6** |
|  | Repetition | 4 | **8.0*** | **15.6*** |
|  | Repetition * Time | 8 | 4.2 | **16.0*** |
|  | Residual | 60 | 31.1 | 24.8 |
| Effevtive QY | Treatment effect | 2 | 5.0 | **20.6*** |
|  | Time | 15 | **14.4** | **18.4** |
|  | Repetition | 4 | **21.6*** | **13.7*** |
|  | Repetition * Time | 8 | 8.6 | **23.3*** |
|  | Residual | 60 | 50.5 | 23.9 |
| Red fluorescence fraction | Treatment | 2 | 0.6 |  |
|  | Time | 14 | 23.9 |  |
|  | Repetition | 4 | **37.7*** |  |
|  | Repetition * Time | 8 | 2.5 |  |
|  | Residual | 56 | 35.3 |  |
| Green fluorescence intensity | Treatment | 2 |  | **21.0*** |
|  | Time | 14 |  | 16.9 |
|  | Repetition | 4 |  | **39.3*** |
|  | Repetition * Time | 8 |  | **10.7*** |
|  | Residual | 56 |  | 12.0 |
| Green fluorescence fraction | Treatment | 2 |  | **41.0*** |
|  | Time | 15 |  | 9.2 |
|  | Repetition | 4 |  | **22.8*** |
|  | Repetition * Time | 8 |  | **22.4*** |
|  | Residual | 60 |  | 4.6 |
| Green fluorescence intensity x fraction | Treatment | 2 |  | **33.6*** |
|  | Time | 14 |  | 10.8 |
|  | Repetition | 4 |  | **33.1*** |
|  | Repetition * Time | 8 |  | **17.4*** |
|  | Residual | 56 |  | 5.0 |
| Red fluorescence intensity | Treatment | 2 |  | **31.0*** |
|  | Time | 15 |  | 13.5 |
|  | Repetition | 4 |  | **15.6*** |
|  | Repetition * Time | 8 |  | **21.8*** |
|  | Residual | 60 |  | 18.1 |
