# Supplementary figures and images for: Effects of reduced dissolved oxygen concentrations on physiology and fluorescence of hermatypic corals and benthic algae
Source: PeerJ. 2014 Jan 2;2:e235. doi: 10.7717/peerj.235 (PMC3898309; doi:10.7717/peerj.235)

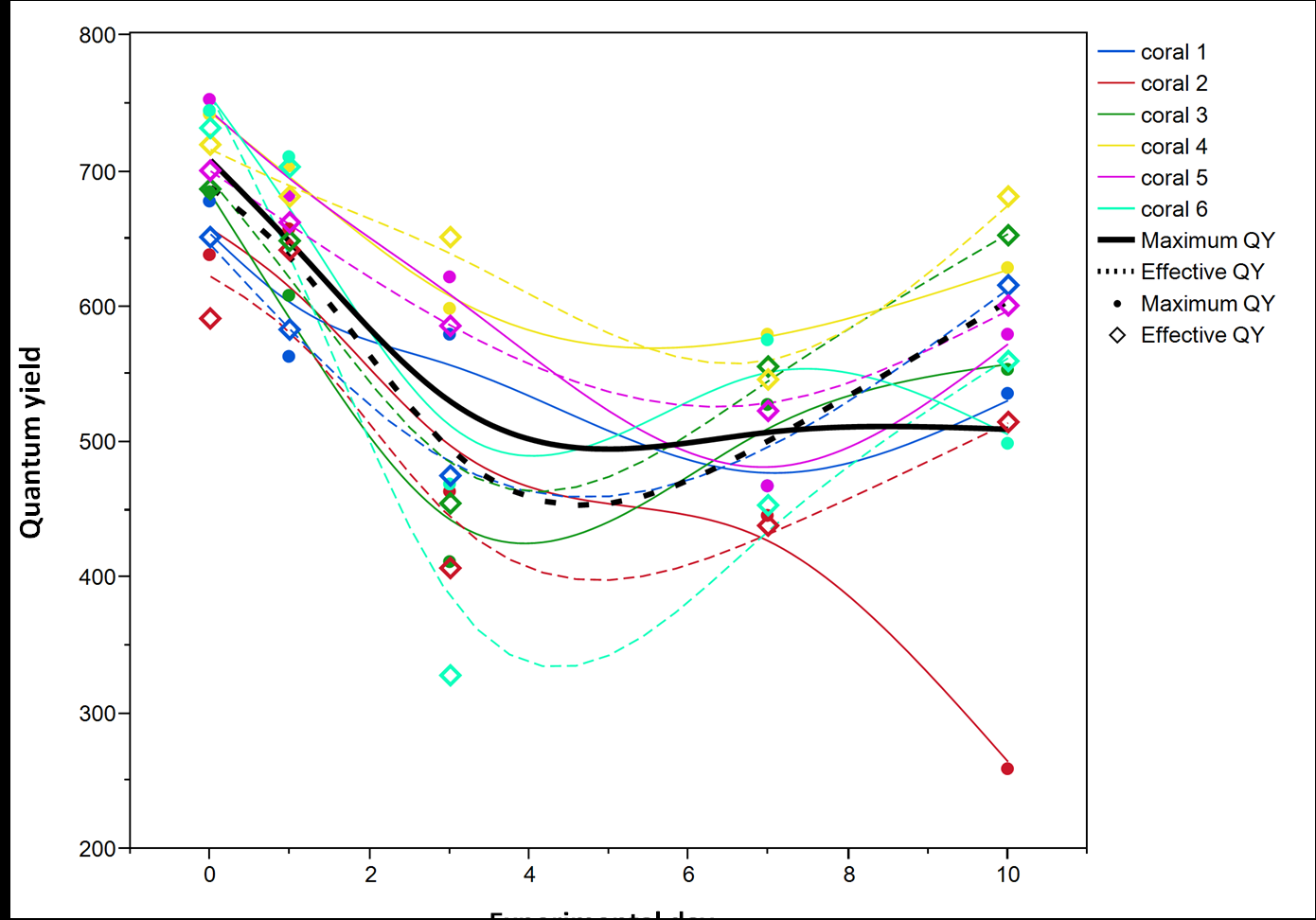

Supplement: Figure S1 — Maximum and effective quantum yield values (QY) for coral specimen subjected to the low oxygen treatment over the course of the experimental period. Individual coral specimens are marked by color while the bold black lines indicate the average effective (dotted lines) and maximum (solid lines) QY values. Note that only on experimental day 10 effective QY values are always higher than maximum QY values. Lines represent a locally weighted scatterplot smoother, i.e. Kernel Smoother. [file peerj-02-235-s003.png]

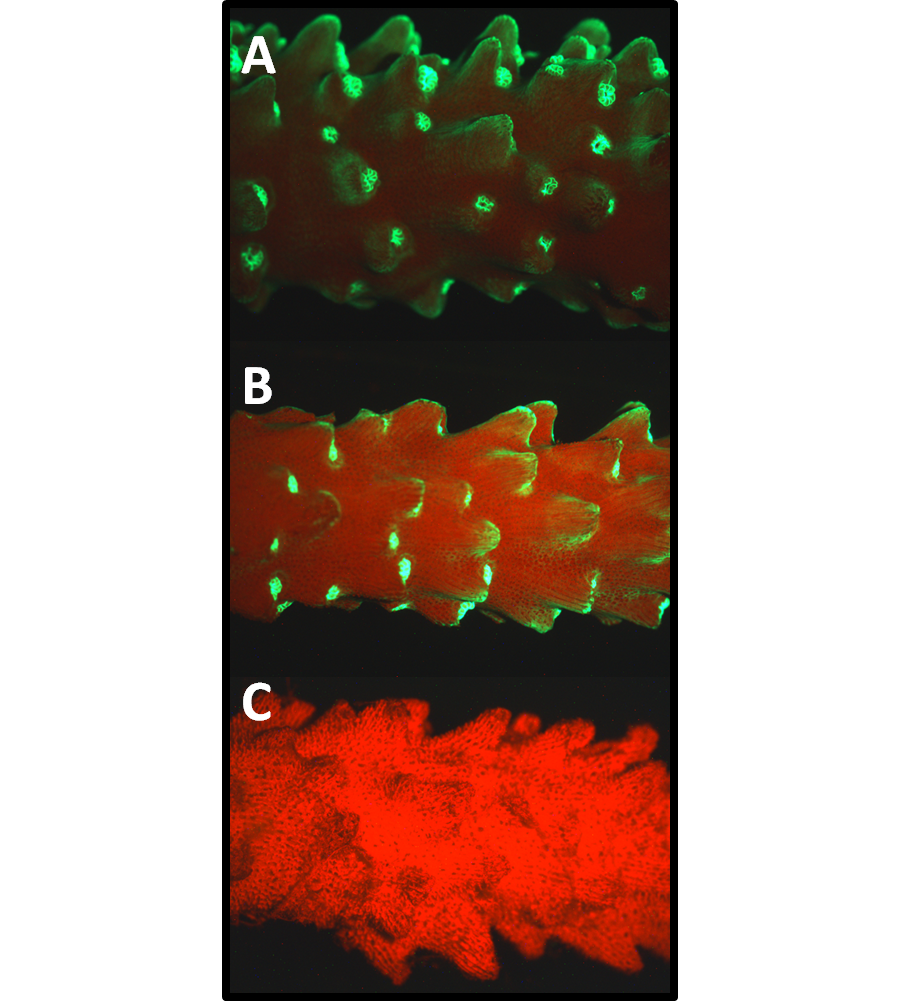

Supplement: Figure S2 — Representative images of the coral A. yongei subjected to (A) 6–8 mg L−1, (B) 4–6 mg L−1, and (C) 2–4 mg L−1 in fluorescence. The pictures visualize the changes in brightness (i.e. amount of GFPs) and distribution (i.e. coenosarc and polyps versus polyps mainly) of green fluorescence. [file peerj-02-235-s004.png]
